# Cervical Cancer Screening Services in Nepal: A Descriptive Cross-sectional Study

**DOI:** 10.31729/jnma.8662

**Published:** 2024-07-31

**Authors:** Bibek Kumar Lal, Ghanshyam Kumar Bhatta, Ramesh Adhikari, Abhishek Karn, K.C Ranju, Sujata Nyaupane, Anuska Adhikari, Binita Shrestha, Merina Shrestha, Pankaj Bhattarai, Sujit Sah, Bijaya Shrestha

**Affiliations:** 1 Family Welfare Division, Ministry of Health and Population, Kathmandu, Nepal; 2 Adventist Development & Relief Agency, Kathmandu, Nepal; 3 Center for Research on Education, Health and Social Science, Kathmandu, Nepal

**Keywords:** *availability*, *cervical cancer*, *Nepal*, *screening*, *survey*

## Abstract

**Introduction:**

Cervical cancer, the most prevalent cancer among women, is also the primary cause of cancer-related deaths in reproductive age women in Nepal. The study aims to assess the situation of cervical cancer screening services in Nepal.

**Methods:**

This cross-sectional study used quantitative methods to understand the situation of cervical cancer screening in 572 health facilities of Nepal. Ethical approval was obtained from the Nepal Health Research Council (NHRC) under reference number 397/2022 P. The research was conducted to assess screening facilities, the allocation of separate screening rooms, the availability of screening services on a routine basis throughout the week, screening facilities that are free of charge, human resources, and the availability of treatment. It was conducted in October to December 2022.

**Results:**

The research was carried out among 572 healthcare facilities, out of which 134 (23.42%) had provision for cervical cancer screening services. Amongst those 134, 72 (53.73%) healthcare facilities had assigned separate rooms for screening intentions. One hundred and two (76.12%) healthcare facilities provided regular screening services throughout the week, while 112 (83.58 %) had free-of-charge screening services. Concerning trained personnel, 121 (90.30%) health facilities had personnel trained in visual inspection with Acetic acid, 9 (6.72%) had personnel trained in use of colposcopy, and 5 (3.73%) had personnel trained in use of Loop Electrosurgical Excision Procedure (LEEP). Lastly, 31 (23.13%) healthcare facilities offered treatment choices for cervical cancer.

**Conclusions:**

Most of the health care facilities did not have provision of cervical screening. Amongst those who had the provision, personnel trained in loop electrosurgical excision procedure colposcopy is lacking.

## INTRODUCTION

Cervical cancer is the fourth most common cancer in women, with approximately 570,000 fresh diagnoses and 311,000 fatalities in 2018.^1^ Early detection of cervical cancer can help in the prevention and treatment of patients.^2^ High-grade cervical precancers can be attributed to the presence of HPV 16 & 18.^3^ Cervical cancer control involves vaccination, screening, treating pre-cancerous lesions, diagnosing invasive cancer, and palliative care.^4^ By 2030, the global strategy aims to achieve specific targets which are, 90% HPV vaccination coverage for young female, 70% screening of women aged 35-45, and proper treatment for 90% of diagnosed cases.^4-6^

In Nepal, guidelines for cervical cancer screening among women aged 30 to 60, aimed target of 70% by 2015.^7^ Only 8.2% of women aged 30-49 years had undergone screening, until 2019.^1^ Regular screening can reduce the number of deaths from cervical cancer.^8^

The study aims to assess the situation of cervical cancer screening services in Nepal. It seeks to identify the details of health facilities, screening services, and the time, cost, and treatment options available in the health facilities of Nepal.

## METHODS

A facility-based descriptive cross-sectional study was conducted from October to December 2022 in the selected health facilities from all seven provinces of the country. The quantitative component of the study involved using a structured questionnaire administered face-to-face with the health facility in-charge to assess the facility.

Nepal is a federal republic comprising seven provinces. Each province consists of 8 to 14 districts, which are further divided into local units known as urban and rural municipalities. This research was conducted across all provinces and included 26 districts: four districts each from Gandaki, Bagmati, Koshi, Madhesh, and Lumbini provinces, and three districts each from Karnali and Sudurpashchim provinces. The allocation was varied because Karnali and Sudurpashchim have fewer districts compared to the other provinces. The selected districts were representative of all geographic areas in Nepal, including the mountain, hill, and Terai regions. A random sampling method was used to select the districts, covering approximately one-third (33.67%) of all districts in Nepal. As a result, the study can be considered nationally representative.

The sample size for each category of Serivce Delivery Points (SDPs) was calculated based on the formula suggested by United Nations Population Fund (UNFPA) guideline.^9^


$$\text{n}={\text{Z}}^{\text{2}}\frac{\text{p}\left(\text{1-p}\right)}{{\text{d}}^{\text{2}}}$$

Where

n = Minimal sample size for each domain
Z = Z score that corresponds to a confidence interval (i.e. 3.84)
p = the proportion of the attribute SDPs expressed in decimal (Tertiary level=0.004, Secondary level 0.0284, PHCC 0.047, UHC 0.101 and Health post=0.823)
d= percent confidence level in decimal (0.05)

The following steps were used to reach the final sample size.

Step 1: Design a sample frame

Step 2: Calculation of relative proportion for different types of SDPs

Step 3: Apply the formula to obtain a minimal sample size for each type of SDPs

Step 4: Distribution of sample sizes for administrative units

Step 5: Sample size adjusted with design effect

According to the Department of Health Services (DoHS) Annual report for the fiscal year 2075/76 (2018), a total of 1,713 public and Non-Governmental Organization (NGO)-run health facilities exist in the selected districts. Using the formula and applying the steps mentioned earlier, with a design effect of 1.1, 572 health facilities were selected for the study.

All the tertiary and secondary level health facilities in the sampled study districts, as well as all NGO-run health facilities providing family planning services (such as Family Planning Association Nepal and Marie Stopes Centers), were included in the study using the census method. For primary-level health facilities (PHCC, Health Post, and Urban Health Clinic), a systematic random sampling technique was used to select them.

Then, a sampling interval for PHCC, Health post and Urban Health Clinic was determined using the formula

i= N/n

Where,

i = Sampling interval for the domain (3.6)
N = Number of SDPs in the domain (1673)
n = Sample size for the domain (475)

The starting health facility (first health facility = k) was selected randomly from a number between 1 and the sample interval. Then, other health facilities were selected by adding the sample interval up to the required sample size from the domain.

For the quantitative survey, a questionnaire prepared by UNFPA was used.^9^ The questionnaires were discussed with the experts from Family Welfare Division (FWD), Adventist Development and Relief Agency (ADRA), and UNFPA, and necessary changes were made.

Seven supervisors were assigned to each province. A team of at least two researchers was deployed in each survey district. One team was responsible for covering the entire sample in one district, and in some cases where they had to cover more than one district, the team had more than two researchers. The supervisor's role was to ensure the quality of data at the field level.

The data collection was conducted digitally using Open Data Kit (KOBO/ODK). The statistician, team leader, and team members cross-checked and ensured the data's quality during the data cleaning and analysis process. Any inconsistencies identified in the data collection procedures were noted and verified in consultation with the enumerators.

The data was then downloaded into Excel sheets, where it was cleaned and coded. The final data was transferred to the Statistical Package for Social Science (SPSS) version 26 (IBM Corp., Armonk, NY) for analysis. An analysis was performed based on federal and provincial disaggregation of the health facilities (UHC, HP, PHCC, Hospitals etc) and their types (public or NGO run).

A total of 50 researchers were hired for data/information collection, consisting of 7 provincial-level supervisors and 43 enumerators. The selection of researchers was based on their education level and experience.

Ethical approval was obtained from the Nepal Health Research Council (NHRC) under reference number 397/2022 P. The study teams strictly adhered to existing social and research ethics. In addition, the core team of Center for Research on Education Health and Social Science (CREHSS) and a representative from NHRC provided orientation on ethical considerations to all the researchers during the training sessions. Research ethics were followed throughout the study period.

## RESULTS

A comprehensive overview of the 572 health facilities selected for the study was assessed, out which 134 (23.42%) had cervical cancer screening facilities. Among the total surveyed health facilities, 482 (84.26%) were primary-level Service Delivery Points (SDPs), 51 (8.92%) secondary-level SDPs and 14 (2.45%) tertiary-level SDPs. The availability of cervical cancer screening services were as follows: 11 (78.57%) in tertiary level SDPs as per level of service delivery point, 14(100%) in FRAN as per types of SDPs, 24 (96%) in NGO as per managing authorities, 71 (21.8%) in hilly areas as per ecological region, 96 (27.43%) in urban area as per location of SDPs and 30 (47.62%) in Gandaki province (Table 1).

**Table 1:** Availability of cervical cancer screening services (N= 572).

	Count	Yes n (%)
**Level of Service Delivery Point**		
Primary Level SDPs	482 (84.26)	71 (14.73)
Secondary Level SDPs	51 (8.92)	28 (54.9)
Tertiary Level SDPs	14 (2.45)	11 (78.57)
NGO Health Facility	25 (4.37)	24 (96)
**Type of SDPs**		
Health Post	248 (43.36)	33 (13.31)
PHCC	70 (12.24)	20 (28.57)
UHC	164 (28.67)	18 (10.98)
District Hospital	16 (2.80)	9 (56.25)
Urban/other hospital	35 (6.12)	19 (54.29)
Federal/regional/Province hospital	13 (2.27)	10 (76.92)
Academia	1 (0.17)	1 (100)
FPAN	14 (2.45)	14 (100)
Marie Stopes Center	11 (1.92)	10 (90.91)
**Managing Authority**		
Public	547 (95.62)	110 (20.11)
NGO	25 (4.38)	24 (96)
**Ecological region**		
Mountain	68 (11.90)	7 (10.29)
Hill	238 (41.60)	71 (29.83)
Terai	266 (46.50)	56 (21.05)
**Location of SDPs**		
Urban	350 (61.19)	96 (27.43)
Rural	222 (38.81)	38 (17.12)
**Provinces**		
Koshi Province	73 (12.76)	31 (42.47)
Madhesh Province	103 (18.01)	14 (13.59)
Bagmati Province	132 (23.08)	23 (17.42)
Gandaki Province	63 (11.01)	30 (47.62)
Lumbini Province	89 (15.56)	14 (15.73)
Karnali Province	50 (8.74)	7 (14)
Sudurpaschim Province	62 (10.84)	15 (24.19)

SDPs: Service Delivery Point, PHCC: Primary Health Care Center, UHC: Urban Health Clinic, FPAN: Family Planning Association Nepal, NGO: Non-Governmental Organization, N: Total Number of Health Facilities, n: Types of health facilities screened

Among the health facilities with availability of cervical cancer screening services, 72 (53.73%) had a designated separate room for cervical cancer screening, 102 (76.12%) had availability of cervical cancer screening all-round the week, and 112 (83.58%) offered free cervical cancer screening, (Table 2).

**Table 2:** Availability of separate room for cervical cancer screening service, services all-round the week, and free-of-charge services (N = 572).

		Availability of separate room for cervical cancer screening service	Regular availability of cervical cancer screening service all-round the week	Availability of cervical cancer screening service free of cost
		n (%)	n (%)	n (%)
**Level of Service delivery point**	Primary Level SDPs (n=71)	27 (38.03)	50 (70.42)	70 (98.59)
	Secondary Level SDPs (n=28)	13 (46.43)	19 (67.86)	27 (96.43)
	Tertiary Level SDPs (n=11)	8 (72.73)	10 (90.91)	9 (81.82)
	NGO Health Facility (n=24)	24 (100)	23 (95.83)	6 (25)
**Type of SDPs**	Health Post (n=33)	6 (18.18)	20 (60.61)	33 (100)
	PHCC (n=20)	7 (35)	14 (70)	19 (95)
	UHC (n=18)	14 (77.78)	16 (88.89)	18 (100)
	District Hospital (n=9)	6 (66.67)	5 (55.56)	9 (100)
	Urban/other hospital (n=19)	7 (36.84)	14 (73.68)	18 (94.74)
	Federal/regional/Province hospital (n=10)	7 (70)	10 (100)	8 (80)
	Academia (n=1)	1 (100)	-	1 (100)
	FPAN (n=14)	14 (100)	13 (92.86)	4 (28.57)
	Marie Stopes Center (n=10)	10 (100)	10 (100)	2 (20)
**Managing Authority**	Public (n=110)	48 (43.64)	79 (71.82)	106 (96.36)
	NGO (n=24)	24 (100)	23 (95.83)	6 (25)
**Ecological region**	Mountain (n=7)	3 (42.86)	5 (71.43)	7 (100)
	Hill (n=71)	39 (54.93)	55 (77.46)	60 (84.51)
	Terai (n=56)	30 (53.57)	42 (75)	45 (80.36)
**Location of SDPs**	Urban (n=96)	64 (66.67)	70 (72.92)	74 (77.08)
	Rural (n=38)	8 (21.05)	32 (84.21)	38 (100)
**Provinces**	Koshi Province (n=31)	15 (48.39)	23 (74.19)	29 (93.55)
	Madhesh Province (n=14)	7 (50)	8 (57.14)	12 (85.71)
	Bagmati Province (n=23)	16 (69.57)	13 (56.52)	16 (69.57)
	Gandaki Province (n=30)	16 (53.33)	26 (86.67)	27 (90)
	Lumbini Province (n=14)	8 (57.14)	12 (85.71)	11 (78.57)
	Karnali Province (n=7)	4 (57.14)	5 (71.43)	5 (71.43)
	Sudurpaschim Province (n=15)	6 (40)	15 (100)	12 (80)
**Total**		72 (53.73)	102 (76.12)	112 (83.58)

SDPs: Service Delivery Point, PHCC: Primary Health Care Center, UHC: Urban Health Clinic, FPAN: Family Planning Association Nepal, NGO: Non-Governmental Organization, N: Total Number of Health Facilities, n: number of facilites with availability of cervical cancer screening

Among the 134 health facilities providing cervical cancer screening services, 121 (90.3%) had staff trained in VIA screening. Disaggregation based on ecological regions showed that all health facilities located in Mountain regions 7 (100%) and rural areas 35 (92.11%) had VIA-trained staff. Lumbini had 14 (100%) and Bagmati province had 21(91.3%) health facilities with VIA-trained staff, (Table 3).

The results of the study indicate that cryotherapy or thermos-coagulator treatment is available in 31 (23%) of SDPs. These findings are related to the availability of treatment and the level of SDPs, which have been classified into primary level 3 (4.2%), secondary level 10 (35.7%), tertiary level 4 (36.4%), and NGO-run facilities 14 (58.3%).

The data also presents the distance between the health facilities providing cervical cancer screening services and the nearest warehouse/store where cryotherapy or thermos-coagulator is available. It shows that a greater number of health facilities providing cervical cancer screening services are located within a 9 km range from the nearest warehouse or store.

**Table 3:** Availability of cervical cancer trained staff among the health facilities having cervical cancer screening services (N= 572).

		Availability of the VIA-trained staff	Availability of the Colposcopy trained staff	Availability of the LEEP-trained staff
		n (%)	n (%)	n (%)
**Level of Service delivery point**	Primary Level SDPs (n=71)	62 (87.32)	-	-
	Secondary Level SDPs (n=28)	25 (89.29)	2 (7.14)	1 (3.57)
	Tertiary Level SDPs (n=11)	11 (100)	5 (45.45)	3 (27.27)
	NGO Health Facility (n=24)	23 (95.83)	2 (8.33)	1 (4.17)
**Type of SDPs**	Health Post (n=33)	27 (81.82)	-	-
	PHCC (n=20)	17 (85)	-	-
	UHC (n=18)	18 (100)	-	-
	District Hospital (n=9)	9 (100)	2 (22.22)	1 (11.11)
	Urban/other hospital (n=19)	16 (84.21)	-	-
	Federal/regional/Province hospital (n=10)	10 (100)	4 (40)	2 (20)
	Academia (n=1)	1 (100)	1 (100)	1 (100)
	FPAN (n=14)	13 (92.86)	1 (7.14)	-
	Marie Stopes Center (n=10)	10 (100)	1 (10)	1 (10)
**Managing Authority**	Public (n=110)	98 (89.09)	7 (6.36)	4 (3.64)
	NGO (n=24)	23 (95.83)	2 (8.33)	1 (4.17)
**Ecological region**	Mountain (n=7)	7 (100)	2 (28.57)	1 (14.29)
	Hill (n=71)	65 (91.55)	3 (4.23)	1 (1.41)
	Terai (n=56)	49 (87.5)	4 (7.14)	3 (5.36)
**Location of SDPs**	Urban (n=96)	86 (89.58)	9 (9.38)	5 (5.21)
	Rural (n=38)	35 (92.11)	-	-
**Provinces**	Koshi Province (n=31)	30 (96.77)	1 (3.23)	-
	Madhesh Province (n=14)	11 (78.57)	-	-
	Bagmati Province (n=23)	21 (91.3)	3 (13.04)	1 (4.35)
	Gandaki Province (n=30)	26 (86.67)	1 (3.33)	-
	Lumbini Province (n=14)	14 (100)	4 (28.57)	3 (21.43)
	Karnali Province (n=7)	6 (85.71)	-	-
	Sudurpaschim Province (n=15)	13 (86.67)	-	1 (6.67)
**Total**		121 (90.3)	9 (6.72)	5 (3.73)

SDPs: Service Delivery Point, PHCC: Primary Health Care Center, UHC: Urban Health Clinic, FPAN: Family Planning Association Nepal, NGO: Non-Governmental Organization, N: Total Number of Health Facilities, n: number of facilites with availability of cervical cander screening

## DISCUSSION

In our study, we found that only 134 (23.42%) of healthcare facilities surveyed offered cervical cancer screening services, with the majority of these facilities being district and tertiary centers. This finding provides one of the important cause for low cervical screening in Nepali women as per the research conducted by Bruni and colleagues, which revealed that only 2.8% of Nepali women were screened for cervical cancer, despite a population of 11.4 million women aged 15 years and older being at risk.^10^ Similar cases of limited availability of cervical cancer screening and treatment have also been reported in other studies^11,12^ particularly in low- and middle-income countries (LMICs). In contrast, high-income countries tend to have higher availability of cervical cancer screening services. For instance, countries like Thailand have demonstrated cost-effective screening programs that improve individual health outcomes through early detection.^13^ In Nepal, most of the available screening facilities were found in urban and tertiary centers. These figures contradict the World Health Organization's recommendation of achieving a 70% screening rate.^6^

Regarding the provision of separate rooms for screening, only 72 (53.73%) out of 134 healthcare facilities with cervical caner screening facilities had such facilities. Having a separate room for cervical cancer screening is crucial to ensure patient confidentiality and privacy. Many women and girls may feel shy or hesitant to visit healthcare facilities and share their issues related to cervical cancer. The presence of a separate room can alleviate these concerns and encourage more women to utilize screening services. A study conducted in Bangladesh revealed that some patients did not seek screening services due to shyness and lack of awareness about available screening options.^14^ Among the 134 healthcare facilities with cervical cancer screening facilities, 112 (90.3%) offered free cervical cancer screening services, which could significantly influence patient attendance. Similar results were reported in studies conducted in South Africa, Japan, and China, where women visited healthcare facilities more frequently when screening services were provided free of charge.^15,16^ In Korea, cervical cancer screening is conducted free every two years^8^. Despite 112 healthcare facilities offering free screening, treatment services were only available at 31 facilities, limiting their capacity to serve patients.

Treatment services were primarily provided by tertiary and secondary healthcare facilities^17^, reflecting similar patterns of limited treatment availability in low- and middle-income countries (LMICs) compared to high-income countries (HICs).

The availability of trained human resources at the healthcare facilities was also limited. Among the facilities providing cervical screening facilities, 90% had staff trained in VIA screening however staff trained in colposcopy 9 (6.72%) and LEEP 5 (3.73%) were limited. The availability of trained staff is a major issue in many low-income countries where the doctor-to-patient ratio is low^18^. Staff training is often limited which is contrary to the WHO standard.^17^

## CONCLUSIONS

Majority of the health care facilities did not have provision of cervical cancer screening. Amongst those who had the provision of screening, only half of the faculties had separate room for examination however most of the facilities had all round the week and free of cost screening facilities. Though most of the center had VIA trained staff, colposcopy and LEEP trained staffs were limited.
